# Side effects of mandibular advancement devices in obstructive sleep apnea patients – observational results of a randomized controlled trial

**DOI:** 10.1007/s11325-026-03576-4

**Published:** 2026-02-03

**Authors:** Olaf Bernhardt, Nikolaos Nikitas Giannakopoulos, Horst Kares, Alexander Meyer, Carl-Maximilian Nürnberger, Sebastian Ruge, Christian Schwahn, Jörg Schlieper, Sarah Yousif, Hendrik Zenner, Philipp Kanzow

**Affiliations:** 1 Department of Restorative Dentistry, Periodontology and Endodontology, University Medicine Greifswald, W. Rathenaustr. 42 a, 17475 Greifswald, Germany; 2 Department of Prosthodontics, University Clinic of Würzburg, Pleicherwall 2, 97070 Würzburg, Germany; 3 Department of Prosthodontics, National and Kapodistrian University of Athens, Athens, Greece; 4 Private Practice, Im Alten Tal 3, 66386 St. Ingbert, Germany; 5 Private Practice, Friedrich-Ebert-Straße 21, 42719 Solingen, Germany; 6 Private Practice, Erlenring 9, 04821 Brandis, Germany; 7 Department of Prosthetic Dentistry, Gerodontology and Biomaterials, University Medicine Greifswald, W. Rathenaustr 42 a, 17475 Greifswald, Germany; 8 Private Practice, Osdorfer Weg 147, 22607 Hamburg, Germany; 9 Private Practice, Salvador-Allende-Straße 30, 17036 Neubrandenburg, Germany

**Keywords:** Sleep apnea, obstructive, Dental sleep medicine, Mandibular advancement device, Side effects, Clinical procedure

## Abstract

**Purpose:**

To identify side effects of two different mandibular advancement devices (MAD) for treating obstructive sleep apnea.

**Methods:**

Sixty-five patients were randomly assigned to two MADs (MAD 1 with bilateral sliding wings, MAD 2 with bilateral bars according to the Herbst appliance). Instructions for supportive jaw exercises and a morning occlusal guide were supplied. Orofacial pain, tenderness of masticatory muscles and temporomandibular joints, interdental contact analysis (digital bite registration system, Greifswald Digital Analyzing System (GEDAS) and Shimstock-foil), Epworth Sleepiness Scale, Pittsburgh Sleep Quality Index, and the Oral Health Impact Profile 5 were assessed before and after one year of treatment. MADs were compared using linear models, which were adjusted for study center, age, sex, and baseline values. Tooth type and tooth status were also included in the multilevel model of interdental contacts.

**Results:**

This study did not contradict the supposition that both MADs yield the same mean for the primary outcome, orofacial pain (P = 0.107; n = 49), possibly because of the reduced sample size due to the COVID-19 pandemic. Significant loss of posterior tooth contacts (n = 37) was established when applying the digital analysis for both MADs. Somnological variables were decreased in the follow-up examination, with no relevant differences between both MAD designs.

**Conclusion:**

For comparing pain in MAD groups, more data will be needed to pool in meta-analyses. However, in our study, loss of interocclusal contacts in posterior teeth could not be prevented by jaw exercises or the use of an occlusal guide.

ClinicalTrials.gov ID: NCT04050514, 08/01/2019.

## Introduction

Mandibular advancement devices (MADs) are a common therapeutic approach involving appliances for the treatment of snoring and obstructive sleep apnea (OSA) [1]. They are recommended as an alternative treatment option to continuous positive airway pressure (CPAP) therapy, especially in mild to moderate OSA [2]. Several clinical studies have demonstrated the efficacy of MAD therapy, leading its classification as a recommended choice of treatment [2, 3]. Compared with CPAP, MADs have been associated with superior patient adherence [4]. Despite variations in design, all MADs share a comparable mechanism of action based primarily on stabilizing the mandible in a protruded position. This mechanical advancement creates tension in the suprahyoid muscles, promoting dilatation and stabilization of the upper airway, especially at the level of the velum, the tongue base, and the epiglottis [5].

Despite this favorable results, potential side effects may occur during or following the nocturnal use of MADs, depending on the device’s specific design and the patient´s characteristics [6]. At the beginning of treatment, patients may experience hypersalivation, xerostomia, increased dental sensitivity, soft-tissue irritation, or gingival inflammation due to contact with the appliance margins [7]. In the short to medium term, mandibular protrusion may lead to temporomandibular disorders (TMD), including sensations of tension or pain in the masticatory muscles and/or temporomandibular joints (TMJ), which typically subside with continued use [8]. Multiple studies have confirmed that prolonged MAD therapy might lead to measurable occlusal changes in the anterior teeth, most notably a reduction in overjet and overbite [7, 9]. After two years of treatment, in up to 18% of patients the development of a posterior open bite was apparent [10]. Long-term studies indicate that such reduced posterior dentition contacts do not spontaneously resolve over a ten-year period [11]. Jaw exercises are considered as a beneficial strategy for preventing occlusal alterations as well as preventing pain [12].

The aim of the study was to compare two various MAD designs with different protrusion mechanics as well as different interincisal vertical dimensions regarding the extent of harmful side effects of these appliances.

Special attention was laid on examining change of occlusal contacts. We hypothesized that a jaw exercise protocol together with the application of an occlusal guide prevent occlusal changes in the posterior teeth.

## Material and methods

### Subjects

#### Recruitment

Patients were recruited at five dental or maxillofacial surgery clinics that specialized in treating OSA patients with MAD and networked with referring sleep medicine centers. Recruitment took place from December 1, 2019, to December 31, 2021. Follow-up examinations were included in the final analysis till December 21, 2022. The target number of 194 patients, however, could not be reached; only 65 patients were recruited by five of the originally planned nine study centers. This discrepancy was primarily caused due to the global SARS-CoV-2 pandemic. Business closures in various economic sectors or price increases could be possible reasons for not reaching the recruitment target. Due to this major limitation affecting analyses at the subject level (including those of the primary outcome, orofacial pain) we focused on analyses including the tooth level, such as interdental contact analyses. These analyses have greater statistical power than pure subject-level analyses because they have a higher number of observations.

### Sample characteristics

The inclusion criteria for this study were as follows: a medical indication for mandibular protrusion (MAD) due to obstructive sleep apnea (OSA) or a request for snoring treatment; an age of 18 to 75 years; mandible protrusion of at least 5 mm; at least the number of eight teeth or four implants existing per jaw, no or fixed dental restorations or a stable, removable partial denture that occludes at least up to the area of the second premolars on both sides; and mental health, as well as a the signed declaration of informed consent.

### Sample size calculation

The required sample size was determined based on the numerical pain scale (0–10) assuming a difference of 1 point and a standard deviation of 2. This results in an effect size of 0.5 which corresponds to a moderate effect size in terms of Cohen's d. Wilcoxon-Mann–Whitney test was used with a two-sided α = 0.05 and a power of 0.90 assuming a family of logistic distributions (G*Power, version 3.1). The final sample was 156. Assuming the annual dropout rate of 10%, a total sample of 194 was calculated for a two-year period (194-20 = 174 after one year and 174-18 = 156 after two years).

## Study design

This study is part of a randomized multi-center, two-arm trial with an active control group. (ClinicalTrials.gov ID: NCT04050514). Originally, the study aimed to analyze the side effects of two different MADs in 194 OSA and snoring patients over a two-year period.

### Randomization

Randomized grouping into blocks at a ratio of 1:1 took place via sequentially numbered opaque sealed envelopes containing the allocation key for the respective MAD. The assignment was stratified according to sex within the 9 intended study centers. Nine series of 40 pseudo-random numbers for male patients and 20 pseudo-random numbers for female patients were generated with Stata/MP software, version 17.0 (Stata Corporation, College Station, TX, USA) and assigned to the respective patient numbers by a person not participating in the active study phase. Envelopes were sent to the nine primary study centers. Examiners were instructed to open the envelopes after consent of the eligible patients. All study protocols for baseline and follow up examinations were labelled with the individual patient ID including the allocation to the treatment group. The clinical trial coordination center at the University of Greifswald verified the ordered sequence of the patient ID after receiving the study protocols.

## Study procedures

All patients received a structured general and somnological history and physical examination during their first visit to the sleep laboratory. Additionally, stationary polysomnography or outpatient polygraphy was performed. Patients were referred to specialized dental practices for base line dental assessment as well as for the fabrication and integration of a MAD based on a diagnosis at a medical sleep center. There, the number of teeth and fixed as well as removeable restorations, orofacial functional condition according to the Diagnostic Criteria for Temporomandibular Disorders (DC/TMD) and the questionnaire on Graded Chronic Pain (GCPS) [13] and questionnaires for sleep quality as well as oral health related quality of life were recorded at baseline and follow up examination.

Primary outcome was defined as the change of orofacial pain during MAD application, measured on a numeric rating scale (NRS; 0–10) at the time of the follow-up appointments. Occurrence of pain was accounted as largest side effect in MAD treatment with major harm. We asked the following question to the patients: “How would you rate your facial pain right now? Use a scale from 0 to 10, where 0 is no pain and 10 is pain as bad as could be.”

### Following secondary outcomes were assessed

Muscle and temporomandibular joint (TMJ) pain with palpation: We examined m. temporalis (posterior, middle, anterior), m. masseter (origin, body, insertion) TMJ lateral pole and around lateral pole each left and right side resulting in 12 palpation sites for the muscles and 4 palpation sites for the TMJs.

Apnea Hypopnea Index (AHI): AHI is a measure of how many breathing pauses in the form of apneas and hypopneas a patient experiences while sleeping per hour [14].

Oxygen Desaturation Index (ODI): ODI is the number of oxygen desaturations per hour of sleep [14].

Pittsburgh Sleep Quality Index (PSQI): PSQI retrospectively asks for a four-week period the incidence of sleep disturbing events, sleep quality assessment, sleep habits, sleep latency and sleep duration, sleeping medication use, and daytime sleepiness (range 0–21) [15].

Epworth Sleepiness Scale (ESS): The Epworth Sleepiness Scale (ESS) is a short questionnaire for the detection of daytime sleepiness, in which the probability of falling asleep or falling asleep in eight typical everyday situations is retrospectively inquired (range 0–24) [4].

Oral Health Impact Profile (OHIP-5): OHIP is a measurement tool for assessing the oral health-related quality of life in adults. It can describe the individual oral health status of patients and measure and compare disease-related burdens in the course (range 0–20) [16].

The questionnaires (PSQI, ESS, OHIP-5) were completed by the investigators in the form of an interview with the patient. AHI and ODI were collected in the sleep laboratory.

### Occlusion protocol

Bite registrations in habitual occlusion were created using thin, flowing, addition-crosslinking silicone (Greenbite Apple, Detax, Germany) and scanned with a flatbed scanner for evaluation in transmitted light. Layer thicknesses of 20 μm or less were interpreted as occlusal contacts and assigned to the anatomical structures of the occlusal surfaces using the Greifswald Digital Analyzing System (GEDAS). Contacts are given in pixel per tooth (range:0—4139) [17]. Furthermore, the number antagonistic tooth contacts between the upper and lower jaw were recorded with 8 micron-thick Shimstock slides for each tooth. Contacts are given in contacts per tooth (0;1) All occlusal assessments were repeated at least one year after the baseline examination. For both procedures all patients were instructed to close their jaws firmly in maximal intercuspal position. All examiners received detailed instructions and training for the examination procedures according to the study protocol before the study started.

## Materials

### MAD therapy

**Fig. 1:**
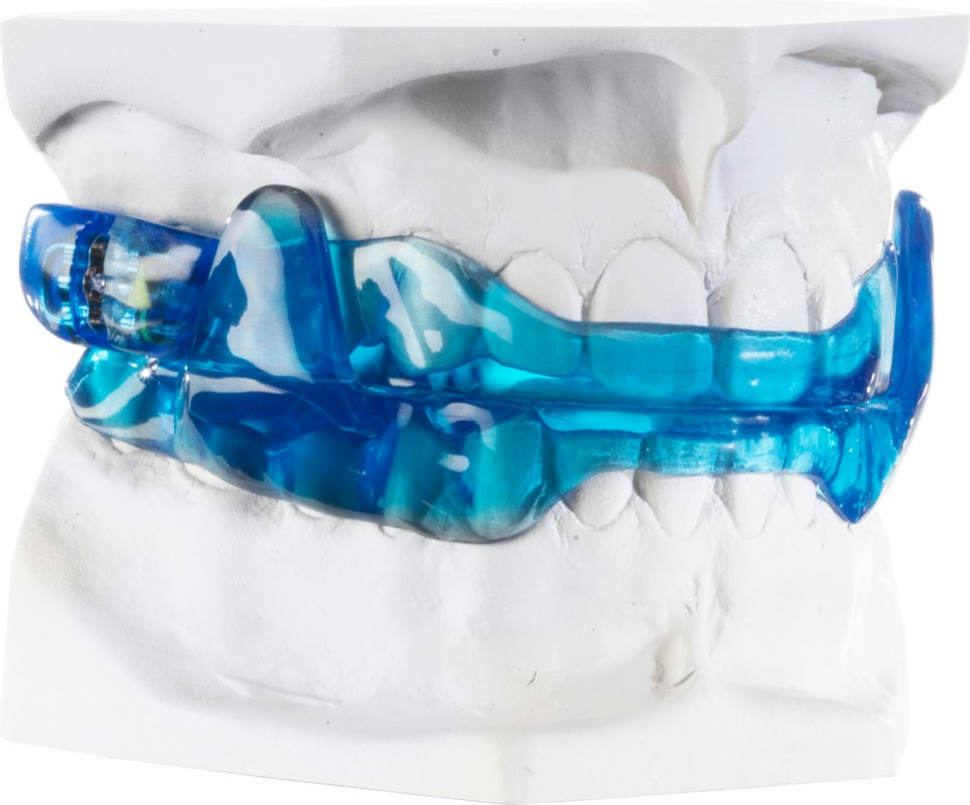
MAD 1

Both included designs of MADs (F-UPS® WO2019238744; H-UPS® DE10216242) were fabricated in the same dental laboratory (Schultheis, Solingen, Germany). MAD 1 was made of polymethyl-methacrylate (PMMA) with bilaterally attached sliding side wings, with a 5-mm interincisal distance, and protrusion as measured by a George Gauge (Great Lakes Dental Technologies, Tonawanda, NY, USA). Lower jaw advancement began at 5 mm from to the maximal possible point of retrusion upon MAD insertion and progressed to the final titrated mandibular advancement during the therapy phase (Fig. 1).

**Fig. 2:**
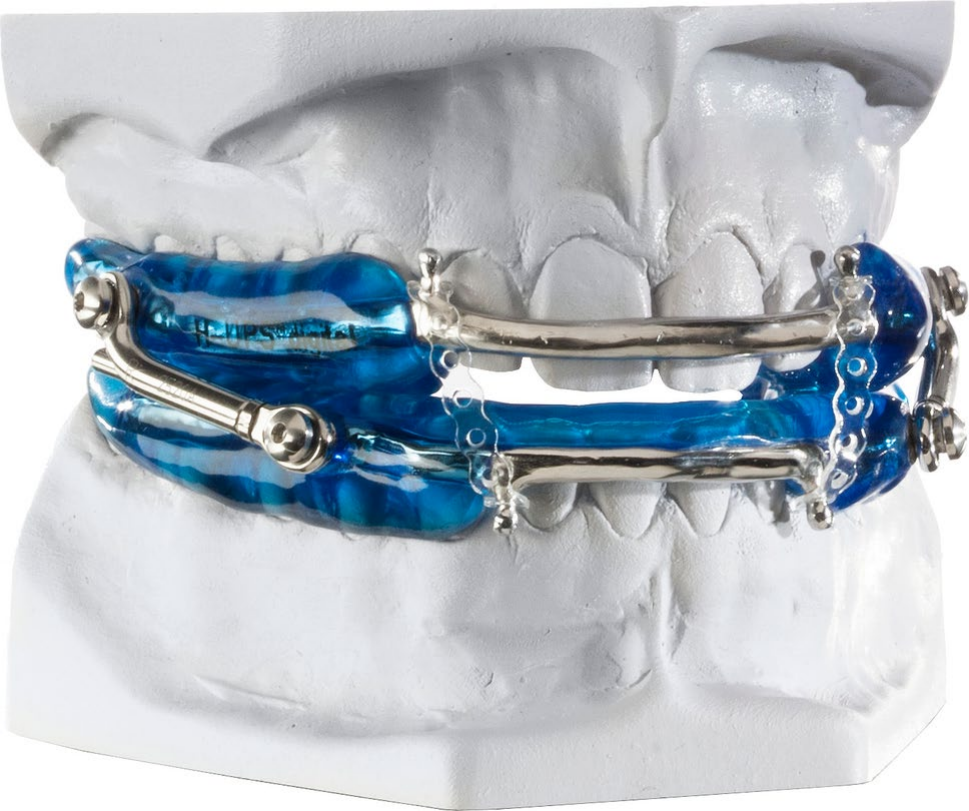
MAD 2

The MAD 2 was made of PMMA including stainless steel-cast inserts with bilateral bars identical to the Herbst appliance, and a 2-mm interincisal distance. Here, due to the steel-cast labial bow, upper incisors had no contact with the appliance. Lower jaw advancement also began at 5 mm protrusion in the same manner as for MAD 1 (Fig. 2).

Due to the different effects of the geometry of the protrusion elements of both devices on the risk of mouth opening, elastics, which can hinder lip closure and comfort and thus the effectiveness of the therapy, were not generally used in MAD 1, in contrast to MAD 2.

After delivery and integration of the assigned MAD, all patients received an extended guide to jaw gymnastics according to Cunali et al. [12] with the instruction to apply the shown exercises in the morning upon waking and in the evening before going to sleep. Patients were as well trained using a morning occlusal guide, the Airway Management Aligner (AM-Aligner, Airway Management Inc., Dallas, TX, USA) which should enable the patients to reestablish occlusion in the habitual pre-treatment position.

## Statistical analyses

We addressed multiplicity by specifying the priority ordering of the outcomes in the study protocol as recommended [18].The secondary outcomes were ordered as follows: muscle and joint pain to assess harm alongside the primary outcome, the number of contact points, as well as PSQI, ESS, OHIP-5, AHI, and ODI. Contrary to the protocol, we removed the STOP-Bang questionnaire and the average oxygen saturation during sleep, but we added titration on demand. As the number of contacts were modeled using multilevel models [19] including subject and tooth level, we structured the text slightly differently for readability.

For the subject-level analysis of the primary outcome, orofacial pain as an ordinal variable, the limiting sample size m was clearly less than the total sample size n [20]. This did not allow for ordinal regression specified in the protocol. To strike a balance between the limiting sample size m (m = 16 for the primary outcome), the guidelines’ requirements, the smallest possible deviation from the study protocol, fulfilling the model assumptions, and ensuring efficiency aspects, we decided to use the linear regression model (m = n = 49 unless stated otherwise). Using splines for continuous covariates addressed the most important mathematical model assumption of the linear regression model. [21, 22]. Nevertheless, we addressed the heteroscedasticity and lack of normality of the residuals using the robust HC3 or Efron variance estimator, which is recommended for small sample sizes [20].

Thus, the linear model allowed for nine degrees of freedom. According to the study protocol, these degrees of freedom were spent for treatment, sex, age, centers, and baseline pain. However, we deviated from the protocol by not including school education in the model. In line with the study protocol, we modeled age using “restricted cubic splines (RCS) with 3 knots, which requires 2 coefficients”. RCS were not applicable for including baseline pain in order to gain efficiency. Instead, we used a linear spline at pain level one. For secondary outcomes on subject-level, we used the same model, but with the corresponding baseline values. However, the baseline values of PSQI and ESS, were modeled using RCS with 3 knots. For titration (n = 34), treatment was adjusted for sex, linear age, and centers (6 degrees of freedom).

The change in the number of pixels between the baseline and one-year follow-up examination was analyzed using linear multilevel models, including 37 patients and 475 teeth combined for both MADs and in separate analyses. The model included group, study center, sex, age, tooth type, and tooth status, as well as the interaction between group and tooth type in additional analyses. Age was modeled using RCS with three knots. Less critical model assumptions, such as homoscedasticity and normality of residuals, showed some deviations[21]. Therefore, small-sample correction according to Kenward and Roger [23] was applied, especially since robust variance estimates are only recommended when at least 42 clusters (patients) are available [19].

Any change in the number of contacts between the baseline examination and the one-year follow-up was analyzed using an ordinal multilevel model for 37 patients and 475 teeth in the combined analysis. The model included the same variables as the model for the analysis of the pixel count. Separate analyses for each MAD were performed accordingly.

**Fig. 3:**
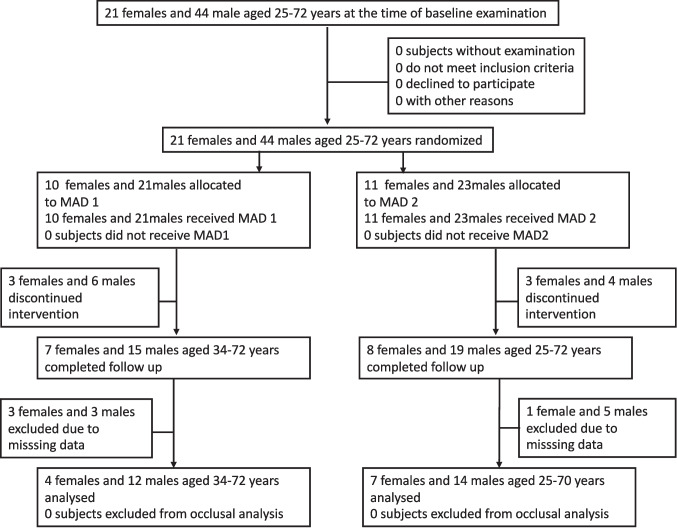
Flow chart of the study population eligible for occlusal analyses, one-year follow up

As only the 16 dropouts (Fig. 3) for whom an outcome was not observed (but no patients for protocol violations) were excluded from the analysis, the analyses presented address the intention-to-treat principle albeit in a less strict interpretation. This principle also applies to the occlusal analysis, as ‘missing at random’ can be assumed for the additional 12 patients with missing occlusal values (Fig. 3) [20]. This was addressed using multilevel models, which can handle ‘missing at random’ data, unlike simple linear regression [20].

The exact Wilcoxon signed-rank test was used for paired observations between baseline and 12 months.

The data were analyzed using Stata/MP software, version 17.0 (Stata Corporation, College Station, TX, USA).

## Results

Of the 65 subjects initially enrolled, 49 patients were available for reevaluation after the period of one year, which was considered the full follow-up period. Sixteen of the subjects dropped out of the study at an early stage. (Fig. 3). A further twelve patients were excluded during data processing due to incomplete data (missing or unusable occlusion protocols) resulting in a sample size of 37 patients for the occlusal analysis.

Demographic characteristics of the 37 patients included in the occlusal contact analyses are presented in Table 1.

**Table 1:** Demographic characteristics of participants of the randomized clinical trial “Occlusal changes after one year MAD treatment in OSA Patients”, n = 37

Variable	MAD 1 (n = 16)	MAD 2 (n = 21)
Age, years Median	52 (44,60)	54 (50,64)
Sex		
Male	12 (75)	14 (67)
Study center		
1	4 (25)	5 (24)
2	8 (50)	11 (52)
3	3 (19)	4 (19)
4	0 (0)	0 (0)
5	1 (6)	1 (5)
Education (highest level)		
< 10 years school	0	1 (5)
10 years school	2(12)	0 (0)
> 10years school	4 (25)	10 (48)
University degree	10 (62)	10 (48)
Marital status		
Married	10 (62)	10 (48)
Married, living separately	0 (0)	2 (10)
Single	4 (25)	6 (29)
Divorced	2 (12)	2 (10)
Widowed	0 (0)	1 (5)
Household income €	4500 (2500–6667)	6667 (2750–6667)
BMI, kg/m^2^	26.2 (24.7–27.9)	25.3 (23.4–28.6)
AHI, episodes, continuously	16.6 (8.6–36.9)	15.9 (11.0–26.0)
AHI episodes, categorical		
0–5	1 (6)	1 (5)
> 5–15	7 (44)	9 (43)
> 15–30	3 (19)	6 (29)
> 30	5 (31)	5 (24)
Max. protrusion	11.5 (10.0–13.0)	10.0 (10.0–12.0)
Tooth number	26 (23–28)	27 (25–28)
Missing teeth	1 (0; 4)	0 (0; 2)
Replaced teeth (FPD	0 (0; 2)	0 (0; 1)
Replaced teeth (RPD)	0 (0; 0)	0 (0; 0)
Pain (NRS)	0 (0; 0)	0 (0; 0)

The number (proportion in the group or column percentage) or the median (interquartile range) is given. BMI: body mass index, FPD: fixed partial denture, AHI: apnoe hypopnoe index RDP: removeable partial denture, NRS: numeric rating scale

### Orofacial pain as the primary outcome

Based on the 95% CI, the data are consistent with a true group difference (MAD 2—MAD 1) in means between −0.06 and 0.57 (point estimate of 0.26; P = 0.107), which is basically a “state of we do not know”. In MAD 1, the single pain level above zero at baseline changed from one to zero after one year, as did the remaining levels (n = 22; P = 1). In MAD 2, four pain levels were above zero at baseline (with a maximum of six), and six pain levels were above zero after one year (with a maximum of three) (n = 27; P = 0.953).

### Secondary outcomes – pain variables

The data for muscle pain with palpation are consistent with a true group difference in means between −0.61 and 0.41 for the 95% CI (point estimate of −0.10; P = 0.685). In MAD 1, the single palpation site at baseline did not occur after one year, as were the remaining subjects without pain (P = 1). In MAD 2, three subjects had at least one palpation site at baseline (with a maximum of six sites in one subject), and three subjects had at least one palpation site after one year (with a maximum of 12 sites in one subject) (P = 0.500).

TMJ pain with palpation did not occur after one year. At baseline, no pain occurred in MAD 1. In MAD 2, two subjects experienced pain at three palpation sites (P = 0.500 for the within group comparison).

### Secondary outcomes – sleep related variables and OHIP

The group difference was 0.88 for the PSQI (95% CI: −0.54 to 2.30; P = 0.216; n = 47), −0.17 for the ESS (95% CI: −1.69 to 1.35; P = 0.818; n = 47), and 0.42 for the OHIP (95%: −0.49 to 1.32; P = 0.357; n = 47). The sample size was too small for modeling AHI and ODI (n = 22 and n = 18, respectively).

For the PSQI the observed values were 5 (4–8) at baseline and 4 (3–6) after one year (P = 0.035; n = 47). For the ESS the corresponding values were 6 (3–9) and 5 (3–7) (P < 0.001; n = 47).

For the OHIP, the corresponding values were 0 (0–2); and 0 (0–2), P = 0.677; n = 47). Overall a reduction in the AHI from 14 (10–24.1) events per h to 4.6 (2–7.4) events per h was observed (P < 0.001; n = 22). There was also a decrease in the ODI (n = 18) from 10.3 (0–19.9) to 2.9 (0.8–6.6) (P = 0.001; n = 18).

### Titration

Regarding final length of the lower jaw advancement the modeled treatment difference was −0.39 (95% CI: −0.97 to 0.18; P = 0.171; n = 34), with observed values of 5.25 mm (5–6.5) and 5 mm (5–5) in groups 1 and 2, respectively.

### Secondary outcomes – occlusal variables

Table 2 shows the median and interquartile range of the pixels that represent the contact areas of the occluding teeth determined by the GEDAS at baseline and at one year follow up. Pixels are calculated per tooth type of the upper jaw and summed up for the left and right sides.

**Table 2:** Pixel count (according to image resolution of 300 dpi) of occluding teeth (right and left sides pooled) determined by GEDAS at baseline and one year follow up for 475 observations/teeth (37 patients) MAD 1and 2 combined and separate

	Combined MAD 1 + MAD 2				MAD 1				MAD 2			
	Baseline		One year follow up		Baseline		One year follow up		Baseline		One year follow up	
Tooth type (FDI notation)	Median	IQR	Median	IQR	Median	IQR	Median	IQR	Median	IQR	Median	IQR
1	30.5	0–187.8	16	0–142.2	35	0–180.5	0	0–112	19	0–237.5	16	0–228.5
2	35.0	0–157	10	0–82	35	0–135	2	0–76.5	32.5	0–156.8	14	0–101.2
3	117.0	0–312	77	0–209	112	0–181.5	86	0–187	175.5	0–444	71	0–246
4	458.0	134–769	219	3–525	258.5	47.5–471.2	173.5	0–378.8	663	250–877.5	350	72.5–720.5
5	502.0	80.5–953.5	194	1.5–500.5	278	20.5–619	166	10–342.5	736.5	189.2–1247.8	212.5	0–668.2
6	643.0	113–1705	429	146–955	396	36–1155.5	365	70.5–686.5	1343	160–2234	669	245–1393
7	717.0	132–1536	493	60–1073.5	717	310–1232	470	155–1006	768	8.75–2641	495.5	47.5–1211.2

FDI: Federation Dentaire Internationale, IQR: interquartile range

Table 3 shows the total number of contacts between the occluding teeth, as determined by Shimstock-foil in maximal intercuspidation at baseline and the one-year follow-up. Contacts are given for teeth in the upper jaw per tooth type summarized for the right and left side.

**Table 3:** Number of contacts of occluding teeth (right and left sides pooled) determined by Shimstock-foil in maximal intercuspidation at baseline and one year follow up for 475 observations/teeth (37 patients), MAD 1and 2 combined and separately

	Combined: MAD 1 + MAD 2 N = 475 (37 patients)				MAD 1 N = 207 (16 patients)				MAD 2 N = 268 (21 patients)			
	Baseline		One year follow up		Baseline		One year follow up		Baseline		One year follow up	
Tooth type (FDI notation)	No contact	Contact	No contact	Contact	No contact	Contact	No contact	Contact	No contact	Contact	No contact	Contact
1	31	39	25	45	13	18	11	20	18	21	14	25
2	35	34	24	45	17	14	11	20	18	20	13	25
3	28	40	15	53	13	18	10	21	15	22	5	32
4	10	59	9	60	6	24	5	25	4	35	4	35
5	9	62	14	57	5	26	5	26	4	36	9	31
6	9	60	14	55	6	22	5	23	3	38	9	32
7	5	54	10	49	2	23	2	23	3	31	8	26
Total	127	348	111	364	62	145	49	158	65	203	62	268

FDI: Federation Dentaire Internationale

Table 4 shows the changes in the number of pixels between the baseline and the one-year follow up, as determined with the GEDAS system in combined and separate analyses for MAD 1 and MAD 2. A linear regression model is presented for each tooth type summarized for the right and left sides, with the first incisor serving as the reference. Third molars were excluded from the analysis. This analysis corresponds to an observational study because tooth type, rather than treatment, was the exposure of interest. There was no evidence against the hypothesis of zero pixel change for the second incisor, canine and first premolar (P > 0.05). A significant drop in pixels was observed in the second premolar, first molar, and second molar (P < 0.05).

**Table 4:** Linear multilevel model for changes in number of pixels (right and left sides pooled) between baseline and one year follow up. Combined and separate analyses for MAD 1 and MAD 2, coefficients, 95% confidence intervals (CI) and P values are given

	Combined: MAD 1 + MAD 2 N = 475 (37 patients)			MAD 1 N = 207 (16 patients)			MAD 2 N = 268 (21 patients)		
Variable	Coefficient	95% CI	*P* value	Coefficient	95% CI	*P* value	Coefficient	95% CI	*P* value
Tooth type (FDI notation)									
1 reference	-	-	-						
2	−15.4	−199.4 – 168.5	0.869	−1.9	−256.8 – 252.9	0.988	−32.9	−269.3 – 230.6	0.806
3	−55.7	−240.6 – 129.1	0.554	31.0	−223.7 – 285.8	0.810	−125.4	−390.5 – 139.8	0.352
4	−102.9	−291.9 – 85.0	0.285	−13.8	−278.8 – 250.4	0.918	−154.0	−423.9 – 115.9	0.262
5	−265.7	−449.7 – −81.8	0.005	−86.5	−344.9 – 171.9	0.509	−410.3	−671.0 – −149.6	0.002
6	−412.3	−508.6 – −226.1	< 0.001	−367.5	−637.3 – 97.6	0.007	−466.4	−726.2 – −206.6	0.001
7	−388.8	−583.6 – −194.8	< 0.001	−310.5	−586.8 – 34.2	0.027	−458.2	−732.3 – −184.0	0.001
MAD design	−140.3	−366.4 – 85.7	0.214	-	-	-		-	-

Adjusted for sex, age, tooth status (present, removable replaced, fixed replaced) and, study center, FDI: Federation Dentaire Internationale

The global (joint) tests over tooth type were significant for both designs (MAD 1: P = 0.018, MAD 2: P < 0.001). MAD design, included as a confounder, showed an uncertain effect (95% CI: −366.4 to 85.7, P = 0.214). Separate analyses revealed a significant change for the second premolar in MAD 2 but not in MAD 1. Furthermore, the interaction between MAD design and tooth type showed a large P value of 0.630, thereby supporting the pooled analysis.

Table 5 shows the ordinal regression model for changes in the number of interdental contacts from baseline to the one-year follow up determined with Shimstock-foil. In the combined analysis, the second incisors and canines showed an insignificant tendency for an increase of contacts, whereas second premolars and molars displayed significantly fewer contacts after one year. Except for tooth status (present teeth or fixed restorations, P < 0.05), none of the other confounders reached significance.

**Table 5:** Ordinal multilevel model for changes in number of interdental contacts (right and left sides pooled) between baseline and one year follow up. Combined and separate analyses for MAD 1 and MAD 2, coefficients, 95% confidence intervals (CI) and P values are given

	Combined MAD 1 + MAD 2 N = 475 (37 patients)			MAD 1 N = 207 16 patients			MAD 2 N = 268 (21 patients)		
Variable	Coefficient	95% CI	*P* value	Coefficient	95% CI	*P* value	Coefficient	95% CI	*P* value
Tooth type (FDI notation)									
1 reference	-	-	-						
2	0.43	−0.44 – 1.29	0.572	0.76	−0.54 – 2.06	0.254	0.14	−1.01 – 1.30	0.806
3	0.62	−0.24 – 1.48	0.157	0.19	−1.14 – 1.54	0.775	0.87	−0.25 – 1.99	0.128
4	−0.85	−1.76 – 0.05	0.066	−1.11	−2.54 – 0.31	0.126	−0.55	−1.77 – 0.65	0.368
5	−1.21	−2.11 – −0.32	0.008	−1.06	−2.46 – 0.33	0.137	−1.45	−2.65 – −0.25	0.017
6	−1.24	−2.15 – −0.34	0.007	−0.83	−2.30 – 0.64	0.268	−1.59	−2.79 – −0.40	0.009
7	−1.25	−2.20 – −0.30	0.010	−0.95	−2.44 – 0.53	0.208	−1.56	−2.82 – −0.30	0.015
MAD design	−0.11	−0.90 – 0.68	0.787	-	-	-	-	-	-

Adjusted for sex, age, tooth status (present, removable replaced, fixed replaced), and study center, FDI: Federation Dentaire Internationale

In separate analyses, MAD 1 in contrast to MAD 2, did not reveal a significant drop of contacts in premolars and molars, MAD 1 and MAD 2, however, showed small P values for the global (joint) tests over tooth type (MAD 1 P = 0.102; MAD 2, P < 0.001). According to modern interpretations of P values, it is not advisable to consider a difference between ‘significant’ and ‘not significant’ as significant in itself [24]. Furthermore, the interaction between MAD design and tooth type showed a large P value of 0.573, thereby supporting the pooled analysis.

## Discussion

We did not record relevant levels of orofacial pain at baseline or in the follow up examination in both MADs. Due to these low pain levels and the low sample size, analysis of group differences resulted in a state of we don’t know. The retention of low levels or just transient increase of orofacial pain and tenderness in the masticatory muscles and the TMJ, however, was described previously [25]. In general, TMD-like symptoms do not appear to worsen with the use of MADs [26].

The effect of both MADs on somnological parameters was similar to that in previous studies [27, 28]. AHI, ODI, ESS and PSQI were significantly reduced. Oral health-related quality of life did not change over time. Improvement of somnological variables indicates that both MADs might have routinely used by the patients.

This is the first study to apply the GEDAS analysis for longitudinal assessment of occlusal alterations in patients treated with MAD for OSA or snoring. After one year of MAD treatment, we observed a measurable decrease in posterior tooth occlusal contacts. None of the confounders included in the combined GEDAS analysis were significant. That means that both MAD designs with 2 mm vs. 5 mm increase in vertical dimension influenced occlusion in similar manner. All patients were trained to perform mandibular exercises in order to prevent pain and maintain posterior contacts [2, 12]. Occlusal side effects, however, could not be prevented.

With exception of the second premolar, separate analyses confirmed the combined analysis determined with the GEDAS system. GEDAS, first reported in 2006, has proven to be a reliable and highly reproducible method for localizing static contacts during intercuspation [17, 29]. It has also been used to identify occlusal contact point patterns in a large-scale study [30].

The results of GEDAS were partly confirmed by interdental contact tests using Shimstock-foil. A lower number of observations in the MAD 1 group may also count for insignificance of posterior contact change. Shimstock-foil, in general suitable for clinical measurements of occlusal contacts in the intercuspal position, provides only a qualitative yes/no decision per tooth pair [31]. It might be not as sensitive for the detection of initial occlusal changes as GEDAS. Further studies with higher power are necessary to investigate this question.

There is strong evidence for dental changes during long-term MAD therapy for OSA patients [9, 32]. Reviews and meta-analyses of randomized controlled trials and non-randomized studies consistently report dento-skeletal remodeling of the intermaxillary relationship. Overbite and overjet decrease mainly due to retroclination of the upper incisors and proclination of the lower incisors. Over time, the lower jaw might undergo clockwise rotation during MAD therapy [9, 32].

Several studies have described infraocclusion in the premolar and molar areas evaluating dental casts after two to ten years of observation [11, 33]. Short-term observation over a period of three months, however, did not establish dental or skeletal changes [28]. In our study we established a loss of posterior contacts yet after one year with digitalized silicone bites and for the MAD 2 also with Shimstock-foil. Posterior open bite might appear immediately after starting the MAD therapy, regardless of preventive interventions, such as jaw exercises. These preventive procedures do not seem to arrest occlusal side effects, and the use of morning occlusal guides to re-adjust mandibular jaw position was not successful in preventing changes in incisor inclination in 23 MAD patients after one year of therapy [34].

Since occlusal changes appeared independent of preventive procedures, a comprehensive education instruction for the patients is necessary. Mostly patients are unaware of small occlusal changes because discomfort due to occlusal discrepancies only occurs after longer periods of time [9]. In the beginning, minor occlusal changes do not seem to be of clinical relevance. At an early stage within the first year of treatment, it is unlikely that patients feel impaired and therapists detect occlusal discrepances, especially if just standard methods as Shimstock-foil are applied. Control appointments with occlusal check-ups should be routinely performed for longer periods. After years of MAD therapy, however, dentists, sleep medicines and patients have to evaluate which effects have a deeper impact on quality of live for the respective patient – changes in occlusion or the consequences of sleep apnea [2].

### Study limitations

The main limitation of the study was the small sample size for the primary outcome, which resulted in deviations from the pre-specified analysis. Main reason for this was the outbreak of the COVID-19 pandemic in Germany at the time of subject recruitment. Only five of the nine study centers were able to contribute patients to the study. Furthermore, MAD therapy was not covered by statutory health insurance at that time, so patients or private insurances had to cover the costs. The pandemic-associated economic crisis in Germany, may have reduced patients' willingness to accept additional financial burdens, further impacting the number of participants. Another drawback, is the inability to measure patient adherence to the exercise and morning occlusal guide protocol, as well as to the MAD. Due to the study setting and the pandemic, it was not feasible to control patient adherence to the training program or employ more expensive types of MAD with integrated micro‐recorder chips. Since tooth type was the focus in the analyses of occlusion, study results have merely observational character. Furthermore, generalizability is limited by the low pain levels observed and the high socio-economic status of the participants. Finally, regression to the mean might also have an influence on measurements [35].

### Strength of the study

Occlusal discrepancies were measured using an innovative digital analysis system based on silicone impressions. This method enabled a blinded analysis of occlusal contact situations [17].

## Conclusions

For comparing pain in MAD groups, more data will be needed to pool in meta-analyses. Regarding the treatment effect for OSA and snoring, both MAD designs were successful in terms of objective and subjective measures (overall reduction of AHI, ODI, ESS and PSQI). Loss of interocclusal contacts in posterior teeth could not be prevented by jaw exercises or the use of an occlusal guide. The GEDAS analysis seems to be suitable for longitudinal tracking of interocclusal contacts.

## Data Availability

Data and materials are available from the contributing author upon request.

## References

[1] Marklund M (2017) Update on oral appliance therapy for OSA. Curr Sleep Med Rep 3:143–151. 10.1007/s40675-017-0080-5 28955651 10.1007/s40675-017-0080-5 PMC5592194

[2] Bernhardt O, Giannakopoulos NN, Heise M, Meyer A, Norden D, Schlieper J, Kares H (2023) Mandibular advancement device: prescription in adult dental sleep medicine - guideline of the German Society of Dental Sleep Medicine. Sleep Breath 27:389–397. 10.1007/s11325-022-02601-6 35349009 10.1007/s11325-022-02601-6 PMC9992253

[3] Phillips CL, Grunstein RR, Darendeliler MA, Mihailidou AS, Srinivasan VK, Yee BJ, Marks GB, Cistulli PA (2013) Health outcomes of continuous positive airway pressure versus oral appliance treatment for obstructive sleep apnea: a randomized controlled trial. Am J Respir Crit Care Med 187:879–887. 10.1164/rccm.201212-2223OC 23413266 10.1164/rccm.201212-2223OC

[4] Arya D, Singh SV, Tripathi A, Tripathi SK (2014) A pilot study to compare patient perception of obstructive sleep apnea treatment with CPAP or appliance therapy. J Prosthet Dent 112:1188–1193. 10.1016/j.prosdent.2014.05.007 24969408 10.1016/j.prosdent.2014.05.007

[5] Guarda-Nardini L, Manfredini D, Mion M, Heir G, Marchese-Ragona R (2015) Anatomically based outcome predictors of treatment for obstructive sleep apnea with intraoral splint devices: a systematic review of cephalometric studies. J Clin Sleep Med 11:1327–1334. 10.5664/jcsm.5198 25979102 10.5664/jcsm.5198 PMC4623132

[6] Sheats RD (2020) Management of side effects of oral appliance therapy for sleep-disordered breathing: summary of American Academy of Dental Sleep Medicine recommendations. J Clin Sleep Med 16:835. 10.5664/jcsm.8394 32105210 10.5664/jcsm.8394 PMC7849794

[7] Fritsch KM, Iseli A, Russi EW, Bloch KE (2001) Side effects of mandibular advancement devices for sleep apnea treatment. Am J Respir Crit Care Med 164:813–818. 10.1164/ajrccm.164.5.2003078 11549538 10.1164/ajrccm.164.5.2003078

[8] de Almeida FR, Lowe AA, Tsuiki S, Otsuka R, Wong M, Fastlicht S, Ryan F (2005) Long-term compliance and side effects of oral appliances used for the treatment of snoring and obstructive sleep apnea syndrome. J Clin Sleep Med 1:143–152 17561628

[9] Rana A, Raut A, Mathur A (2023) The occlusal side effects of mandibular advancement device therapy in adult sleep apnea patients: a systematic review. Cureus 15:e48682. 10.7759/cureus.48682 38090465 10.7759/cureus.48682 PMC10714377

[10] Perez CV, de Leeuw R, Okeson JP, Carlson CR, Li HF, Bush HM, Falace DA (2013) The incidence and prevalence of temporomandibular disorders and posterior open bite in patients receiving mandibular advancement device therapy for obstructive sleep apnea. Sleep Breath 17:323–332. 10.1007/s11325-012-0695-1 22477031 10.1007/s11325-012-0695-1

[11] Uniken Venema JAM, Doff MHJ, Joffe-Sokolova DS, Wijkstra PJ, van der Hoeven JH, Stegenga B, Hoekema A (2020) Dental side effects of long-term obstructive sleep apnea therapy: a 10-year follow-up study. Clin Oral Investig 24:3069–3076. 10.1007/s00784-019-03175-6 31863188 10.1007/s00784-019-03175-6

[12] Cunali PA, Almeida FR, Santos CD, Valdrichi NY, Nascimento LS, Dal-Fabbro C, Tufik S, Bittencourt LR (2011) Mandibular exercises improve mandibular advancement device therapy for obstructive sleep apnea. Sleep Breath 15:717–727. 10.1007/s11325-010-0428-2 20967571 10.1007/s11325-010-0428-2

[13] Schiffman E, Ohrbach R, Truelove E, Look J, Anderson G, Goulet JP, List T, Svensson P, Gonzalez Y, Lobbezoo F, Michelotti A, Brooks SL, Ceusters W, Drangsholt M, Ettlin D, Gaul C, Goldberg LJ, Haythornthwaite JA, Hollender L, Jensen R, John MT, De Laat A, de Leeuw R, Maixner W, van der Meulen M, Murray GM, Nixdorf DR, Palla S, Petersson A, Pionchon P, Smith B, Visscher CM, Zakrzewska J, Dworkin SF, International Association for Dental Research (International RDC/TMD Consortium Network), International Association for the Study of Pain (Orofacial Pain Special Interest Group) (2014) Diagnostic criteria for temporomandibular disorders (DC/TMD) for clinical and research applications: recommendations of the International RDC/TMD Consortium Network* and Orofacial Pain Special Interest Group†. J Oral Facial Pain Headache 28:6–27. 10.11607/jop.1151 10.11607/jop.1151 PMC4478082 24482784

[14] Burlon G, Tepedino M, Laurenziello M, Troiano G, Cassano M, Romano L, Rinaldi R, Ciavarella D (2020) Evaluation of factors that influence the success rate of OSA treatment with a customised adjustable MAD device - a retrospective study. Acta Otorhinolaryngol Ital 40:297–303. 10.14639/0392-100X-N0307 32519991 10.14639/0392-100X-N0307 PMC7586197

[15] Godoy LBM, Palombini L, Poyares D, Dal-Fabbro C, Guimaraes TM, Klichouvicz PC, Tufik S, Togeiro SM (2017) Long-term oral appliance therapy improves daytime function and mood in upper airway resistance syndrome patients. Sleep 40. 10.1093/sleep/zsx175 10.1093/sleep/zsx175 29045745

[16] John MT (2022) Standardization of Dental Patient-Reported Outcomes Measurement Using Ohip-5 - Validation of “Recommendations for Use and Scoring of Oral Health Impact Profile Versions.” J Evid Based Dent Pract 22:101645. 10.1016/j.jebdp.2021.101645 35063174 10.1016/j.jebdp.2021.101645 PMC9123939

[17] Quooss A, Ruge S, Kordass B (2011) GEDAS II–new possibilities in digital contact point analysis. Int J Comput Dent 14:105–109 21877376

[18] Cook RJ, Farewell VT (1996) Multiplicity considerations in the design and analysis of clinical trials. Journal of the Royal Statistical Society Series a-Statistics in Society 159:93–110. 10.2307/2983471

[19] Rabe-Hesketh S, Skrondal A (2022) Multilevel and Longitudinal Modeling Using Stata I, 4th edn. Vol. I: Continuous Responses). Stata Press, College Station

[20] Harrell FE Jr (2015) Regression modeling strategies. With applications to linear models, logistic and ordinal regression, and survival analysis, 2nd edn. Springer, Heidelberg

[21] Gelman A, Hill J, Vehtari A (2021) Regression and other stories. Cambridge University Press, Cambridge

[22] James G, Witten D, Hastie T, Tibshirani R (2021) An Introduction to Statistical Learning, 2nd edn. Springer, New York

[23] Kenward M, Roger JH (2009) An improved approximation to the precision of fixed effects from restricted maximum likelihood. Comput Stat Data Anal 53:2583–2595

[24] Greenland S, Senn SJ, Rothman KJ, Carlin JB, Poole C, Goodman SN, Altman DG (2016) Statistical tests, P values, confidence intervals, and power: a guide to misinterpretations. Eur J Epidemiol 31:337–350. 10.1007/s10654-016-0149-3 27209009 10.1007/s10654-016-0149-3 PMC4877414

[25] Alessandri-Bonetti G, Bortolotti F, Bartolucci ML, Marini I, D’Anto V, Michelotti A (2016) The effects of mandibular advancement device on pressure pain threshold of masticatory muscles: a prospective controlled cohort study. J Oral Facial Pain Headache 30:234–240. 10.11607/ofph.1500 10.11607/ofph.1500 27472526

[26] Alessandri-Bonetti A, Bortolotti F, Moreno-Hay I, Michelotti A, Cordaro M, Alessandri-Bonetti G, Okeson JP (2019) Effects of mandibular advancement device for obstructive sleep apnea on temporomandibular disorders: a systematic review and meta-analysis. Sleep Med Rev 48:101211. 10.1016/j.smrv.2019.101211 31605905 10.1016/j.smrv.2019.101211

[27] Vanderveken OM, Van Daele M, Verbraecken J, Braem MJ, Dieltjens M (2024) Comparative analysis of two custom-made mandibular advancement devices with varied designs for treating moderate to severe obstructive sleep apnea. Sleep Med 117:95–98. 10.1016/j.sleep.2024.02.035 38518588 10.1016/j.sleep.2024.02.035

[28] Yu L, Mao R, Zhang C, Zhao J, Lin N, Sun Z, Zheng Y (2024) Clinical study of two mandibular advancement devices in the treatment of obstructive sleep apnea: a pilot randomized controlled trial. BMC Oral Health 24:1492. 10.1186/s12903-024-05289-0 39696299 10.1186/s12903-024-05289-0 PMC11658169

[29] Hutzen D, Rebau M, Kordass B (2006) Clinical reproducibility of GEDAS–"Greifswald Digital Analyzing System" for displaying occlusal contact patterns. Int J Comput Dent 9:137–142 16955651

[30] Kordass B, Amlang A, Hugger A, Behrendt C, Ruge S (2022) Number and localization of occlusal contact areas on natural posterior teeth without dental findings - evaluations of the regional baseline study (SHIP-1) with the Greifswald Digital Analyzing System (GEDAS). Int J Comput Dent 25:47–56 35322652

[31] Anderson GC, Schulte JK, Aeppli DM (1993) Reliability of the evaluation of occlusal contacts in the intercuspal position. J Prosthet Dent 70:320–323. 10.1016/0022-3913(93)90215-a 8229882 10.1016/0022-3913(93)90215-a

[32] Chen Y, Alhozgi AI, Almeida FR (2024) Dentoskeletal changes of long-term oral appliance treatment in patients with obstructive sleep apnea: a systematic review and meta-analysis. J Prosthodont. 10.1111/jopr.13946 39327689 10.1111/jopr.13946 PMC12000640

[33] Doff MH, Finnema KJ, Hoekema A, Wijkstra PJ, de Bont LG, Stegenga B (2013) Long-term oral appliance therapy in obstructive sleep apnea syndrome: a controlled study on dental side effects. Clin Oral Investig 17:475–482. 10.1007/s00784-012-0737-x 22562077 10.1007/s00784-012-0737-x PMC3579417

[34] Zheng P, Chalidapongse P, Changsiripun C (2023) Mandibular advancement devices used with morning occlusal guides for treating obstructive sleep apnea-changed incisor inclination and its associated factors. Sleep Breath 27:2059–2067. 10.1007/s11325-023-02796-2 36862328 10.1007/s11325-023-02796-2

[35] Barnett AG, van der Pols JC, Dobson AJ (2005) Regression to the mean: what it is and how to deal with it. Int J Epidemiol 34:215–220. 10.1093/ije/dyh299 15333621 10.1093/ije/dyh299

